# PERSONAL: Feasibility Study Protocol for Placebo-Controlled, Randomized n-of-1 Trials of Tamsulosin for Lower Urinary Tract Symptoms

**DOI:** 10.3389/fdgth.2020.00007

**Published:** 2020-06-26

**Authors:** Scott R. Bauer, Benjamin N. Breyer, Akinyemi Oni-Orisan, Michael A. Steinman, Ida Sim, Charles E. McCulloch, Stacey A. Kenfield

**Affiliations:** ^1^Division of General Internal Medicine, Department of Medicine, University of California, San Francisco, San Francisco, CA, United States; ^2^Department of Urology, University of California, San Francisco, San Francisco, CA, United States; ^3^Veterans Affairs Medical Center, San Francisco, San Francisco, CA, United States; ^4^Department of Epidemiology and Biostatistics, University of California, San Francisco, San Francisco, CA, United States; ^5^Department of Clinical Pharmacy and Institute for Human Genetics, University of California, San Francisco, San Francisco, CA, United States; ^6^Division of Geriatrics, Department of Medicine, University of California, San Francisco, San Francisco, CA, United States

**Keywords:** randomized clinical trial design, personalized medicine, patient-reported outcomes, medication side effects, benign prostatic hyperplasia, deprescribing, α-antagonist

## Abstract

**Background:** Lower urinary tract symptoms (LUTS) affect more than half of men over age 70 and contribute to both poor health-related quality of life and polypharmacy. Tamsulosin hydrochloride, a selective α_1_-blocker, is the most common medication used to treat LUTS due to presumed benign prostatic hyperplasia and is often prescribed indefinitely, although not all men benefit from long-term therapy. N-of-1 trials allow for individualized estimates of benefit and harm and could facilitate decisions regarding chronic tamsulosin therapy for LUTS, particularly among older men. Our team developed the PERSONAL (PlacEbo-controlled, Randomized, patient-Selected Outcomes, N-of-1 triALs) app to track daily urinary symptoms and medication side effects for n-of-1 trials among older men with LUTS.

**Materials and Methods:** We will conduct a feasibility study of 20 individual randomized n-of-1 trials using the PERSONAL app to compare tamsulosin (0.4 or 0.8 mg) vs. placebo among older men taking tamsulosin for LUTS. We will include men over age 65 with a smartphone for whom temporary discontinuation of tamsulosin is safe, (e.g., no history of acute retention). Participants will work with research staff to prospectively identify the most important urinary symptoms and medication side effects that they would like to digitally track. Men will then be randomized to 2-week treatment periods of tamsulosin or placebo followed by a 1-week wash-out with placebo, for 4 distinct treatment periods and 3 wash-out periods, totaling 11 weeks. Study medications will be blinded using over-encapsulation of tamsulosin pills and matching placebo. Our primary outcomes for this study will be recruitment and retention of eligible men, completion rates of n-of-1 trials and daily questionnaires using the PERSONAL app, and participants' perceived usefulness of their n-of-1 trial for determining whether tamsulosin is effective for them. Linear mixed effects models with individual-specific intercepts and intervention effects will also be used to estimate within-individual effects of tamsulosin.

**Discussion:** The goal of this innovative study is to establish feasibility and acceptability of using a mobile health app and n-of-1 trials to provide older men with individualized estimates of benefits and harms of chronic tamsulosin therapy for LUTS.

## Introduction

Lower urinary tract symptoms (LUTS), such as nocturia, urinary urgency, and weak stream, affect more than half of men over age 70 (1). LUTS are associated with increased risk of polypharmacy, falls, and psychological distress, all of which contribute to poor health-related quality of life (2–4). Guidelines recommend treating LUTS due to presumed benign prostatic hyperplasia (BPH) with α_1_-blockers (5, 6), which inhibit smooth muscle contraction in the prostate and bladder neck and are increasingly prescribed globally (7–9). Although large randomized controlled trials have demonstrated modest efficacy of α_1_-blockers for improving LUTS severity scores [2.1 to 3.7 point mean difference in the International Prostate Symptom Score (10)], average effect sizes in most individual trials and meta-analyses do not reach the accepted minimally important difference (3 points) (11, 12). These trials also use mean change in LUTS severity scores as the primary outcome, which assumes that all patients use overall symptom severity rather than specific bothersome symptoms to make LUTS treatment decisions (13). Harms of α_1_-blockers, such as orthostatic hypotension and dizziness which lead to falls and fractures, have led to recommendations that they be used with caution in older men (14–16). Unfortunately, alternative LUTS medications, such as 5α-reductase inhibitors, anti-muscarinics, and most recently desmopressin, are also problematic for older men (14, 15, 17). In the setting of modest benefits and known harms, a more personalized and patient-centric approach is needed to ensure that only older men in whom benefits outweigh the harms continue to receive chronic tamsulosin therapy for LUTS.

N-of-1 trials, or multiple crossover trials conducted within a single individual, are a powerful yet underused tool that could be used to optimize prescriptions for symptomatic conditions such as LUTS (18). This study design is particularly well-suited to address a major barrier of deprescribing for both patients and prescribers: the fear of worsening symptoms or complications after stopping a medication that may have provided benefit initially (19, 20). N-of-1 trials carry an additional benefit to older adults given their lack of representation in most rigorous randomized controlled trials, greater heterogeneity in causes of urinary symptoms and response to treatments, and potential for harms from medication side effects and polypharmacy (e.g., adverse drug events and drug-drug interactions) (21). Whereas the trials evaluating efficacy of tamsulosin for LUTS due to BPH were conducted predominantly among relatively healthy white men <65 years old (11), the majority of men who currently receiving chronic tamsulosin therapy are over age 65, have multiple comorbidities, and match the racial diversity in the United States (9, 22). N-of-1 trials can be implemented with or without the involvement of clinicians and are able to accommodate patient-selected outcomes that may be more influential in treatment decisions than overall LUTS severity scores. By leveraging mobile health technology to implement a more personalized approach to prescribing and deprescribing, n-of-1 trials could potentially replace current recommendations to treat bothersome LUTS due to BPH with indefinite α_1_-blocker therapy.

The goal of this study is to establish feasibility and acceptability of using the PERSONAL (PlacEbo-controlled, Randomized, patient-Selected Outcomes, N-of-1 triALs) mobile health app to conduct placebo-controlled n-of-1 trials among older men receiving chronic tamsulosin therapy for LUTS to facilitate deprescribing decisions. This study will include a total of 20 men who will undergo individualized n-of-1 trials in order to collect and report the parameters necessary to plan an optimal and adequately powered full study of drug effectiveness.

## Materials and Methods

### Study Design

Our research team is following a mixed methods approach (23) to develop and evaluate digital health interventions as advocated by the World Health Organization (24). While we plan to conduct focus groups and semi-structured interviews of study participants to further refine the PERSONAL app and study design, the following protocol focuses on the feasibility of conducting placebo-controlled n-of-1 trials among older men with LUTS using a mobile health app.

#### Study Setting

The proposed study will be located within the San Francisco Bay Area with recruitment occurring at multiple clinical sites within the University of California, San Francisco (UCSF) Medical Center. Participants' UCSF clinicians will not be routinely informed of their participation in this study.

#### Study Hypothesis

The primary study hypothesis is that it is feasible to conduct a series of individual placebo-controlled n-of-1 trials among older men receiving tamsulosin for LUTS using PERSONAL app. Specifically, we hypothesize that it is possible to recruit and retain 20 eligible men from a single healthcare system within a reasonable timeframe (e.g., 3–6 months), >70% of enrolled participants will complete n-of-1 trials, participants will complete >50% of daily questionnaires, and >50% of participants will describe the PERSONAL app as “extremely helpful” or “very helpful” for deciding whether tamsulosin is an effective medication for them to continue or discontinue. Secondary and tertiary outcomes will include standardized measures of LUTS severity, global urinary bother, satisfaction with LUTS treatment, attitude toward deprescribing, medication adherence, and health-related quality of life.

#### Eligibility Criteria

Study participants include a broad diversity of patients recruited from the UCSF Medical Center electronic health record (EHR) at 3 UCSF-affiliated clinical sites: Mission Bay Campus, Parnassus Heights Campus, and Zuckerberg San Francisco General Hospital. Participants must meet the following eligibility criteria (Table 1): English speaking men over age 65 with a diagnosis of LUTS or BPH based on International Statistical Classification of Disease and Related Health Problems (ICD-10 codes; N40.1 or R39198); currently prescribed daily tamsulosin therapy [(0.4 or 0.8 mg) for at least the past 12 months; owns an eligible iOS or Android smartphone or tablet; have a Lower Urinary Tract Dysfunction Research Network 10-Item Symptom Index (LURN SI-10(25)] ≤ 10 (corresponds to none/mild to moderate symptoms on a scale of 0 [no symptoms] to 38 [most severe symptoms]). Participants will be excluded if they have urinary incontinence or a condition that requires continuous tamsulosin treatment, such as history of acute urinary retention, recurrent urinary infections, obstructive kidney disease, or ureteral stent. Participants will also be excluded if they have medical conditions, such as dementia or active substance use disorder, that will interfere with their participation in the study.

**Table 1:** Participant inclusion and exclusion criteria.

**Inclusion criteria**	**Exclusion criteria**
Android or iPhone smartphone or tablet with an active data plan and/or connected to a home WiFi network	History of urinary incontinence, acute urinary retention, recurrent urinary tract infections, nephrolithiasis, obstructive kidney disease, urethral stent
Age ≥65 years	Active cancer treatment or medical condition that would limit the patient's life expectancy to <6 months
Diagnosis code for BPH or other micturition problem based on ICD-10 (N40.1 or R39198)	Dementia, bipolar disorder, schizophrenia, active suicidality, active substance use disorder
Taking tamsulosin 0.4 to 0.8 mg daily for ≥12 months	Current participation in another smartphone app-based clinical study
LURN 10-Item Symptom Index (SI-10) ≤ 10 (none/mild to moderate symptoms)	Planning to relocate from study area within 6 months
Downloaded an app from the Google Play or App Store within the past year	Impaired vision that limits the use of smartphone apps
Ability to speak and read English	Unwilling to temporarily stop tamsulosin

*BPH, benign prostatic hyperplasia; ICD, International Statistical Classification of Disease and Related Health Problems; LURN, Lower Urinary Tract Dysfunction Research Network*.

#### Recruitment

**Figure 1:**
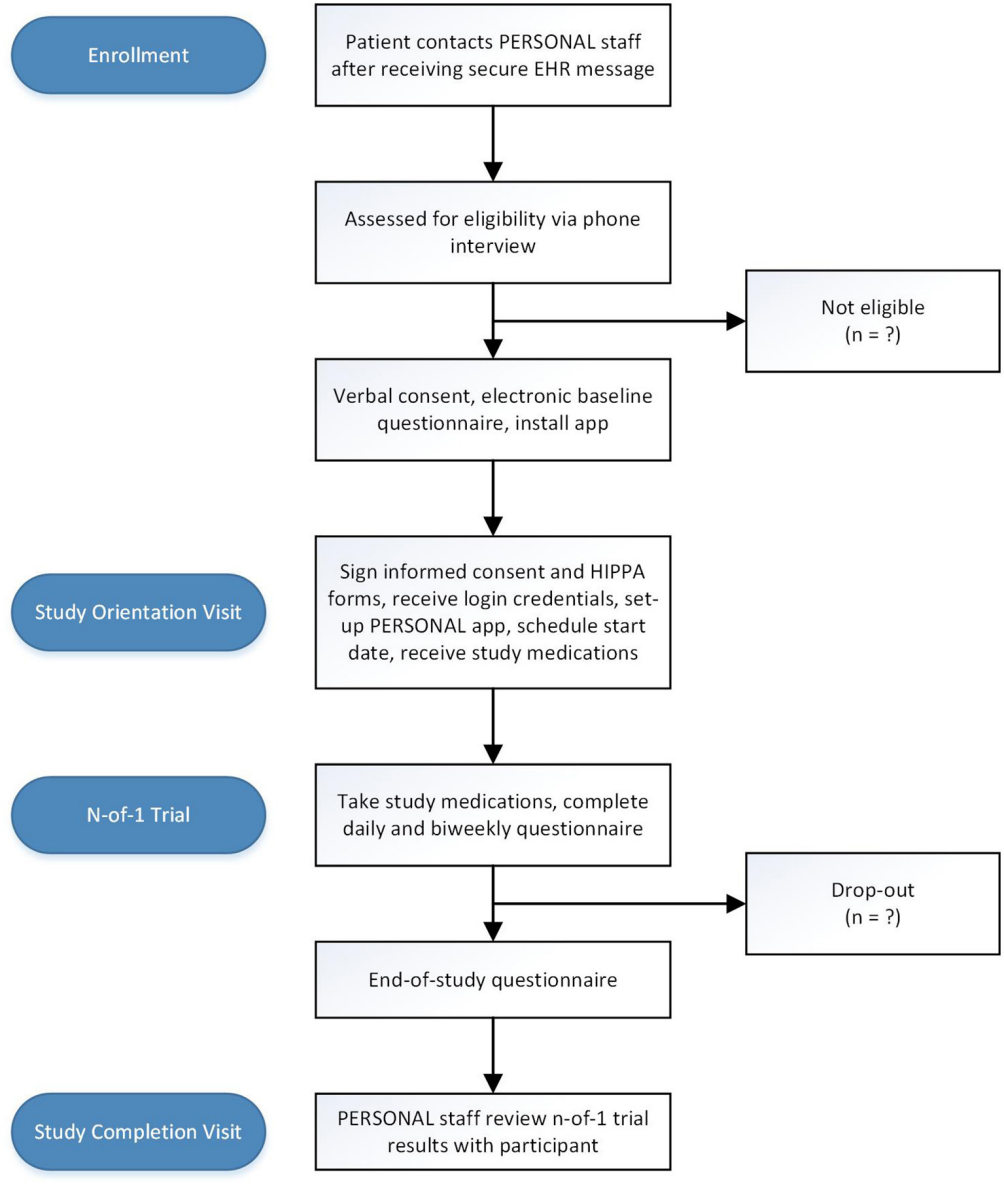
Study flow diagram. EHR electronic health record; IRB institutional review board; HIPPA Health Insurance Portability and Accountability Act.

The participant flow diagram is shown in Figure 1. Patients who meet inclusion criteria based on data available in the EHR and who have previously agreed to be contacted by UCSF Research Participant Services will receive a secure EHR message informing them about the study and inviting them to contact research staff if interested in participating. Patients who have not enrolled in secure EHR messaging will receive a paper-based letter with the same information. Recruitment materials will offer eligible participants $100 for completing the study.

#### Screening

Eligibility will be determined via telephone screening. Research staff will explain the study and ask questions to determine which inclusion and exclusion criteria are met, including whether the patient has an eligible phone device. Once a patient is deemed eligible, they will be asked to schedule a study orientation visit. They will then receive a confirmatory email with a link to complete the baseline questionnaire using Research Electronic Data Capture (RedCap), a secure online portal. They will also receive instructions to download the PERSONAL app (available for free on Google Play and Apple's App Store) on their smartphone prior to the orientation visit.

#### Study Orientation Visit

The orientation visit will be conducted by trained research staff who will first obtain informed consent to participate in the study and a Health Insurance Portability and Accountability Act (HIPPA) authorization form. Research staff will first describe the n-of-1 study design, daily questionnaires, and real-time data visualization and will then ensure successful installation of the PERSONAL app. Each participant will then receive a unique study ID and login credentials for the PERSONAL app. Participants and research staff will customize the app together by selecting symptoms and side effects to track and setting reminder notifications based on the preferences of the participant (e.g., morning reminder if tracking nighttime symptoms or evening reminder if tracking daytime symptoms). The participants will then select a start date for their n-of-1 trial which will be entered directly into the PERSONAL app. Participants will receive a bubble pack with 11 weeks of tamsulosin (at their previously prescribed dose) or matching placebo and will be instructed to start taking the study medications after successfully completing the run-in period. At the end of the orientation visit, research staff will assess understanding of the app features and provide verbal and written instructions for the rest of the study. Research staff contact information will be provided for reporting severe or concerning symptoms or technical app support for the duration of the study.

#### Patient-Selected Outcomes

Participants will be asked at baseline to identify their most bothersome urinary symptoms and perceived medication side effects. Using responses from the baseline questionnaire as a guide, research staff will guide participants to select up to 2 symptoms and up to 2 side effects to track during their n-of-1 study (see Assessments and Outcome Measures below). We will consider allowing participants to track additional symptoms and side effects if they perceive the burden of additional daily questions to be low. Once selected, these outcomes will be entered directly into the PERSONAL app to create a personalized n-of-1 trial focused on the specific outcomes of interest for each participant. If participants cannot identify a preferred symptom or side effect for tracking, this will be recorded, and the default urinary symptom will be their most frequent symptom identified on the baseline questionnaire. The default medication side effect will be dizziness/lightheadedness because it is one of the most common (15% to 17%) and serious side effects of tamsulosin among older men (26).

#### N-of-1 Trial Description

**Figure 2:**
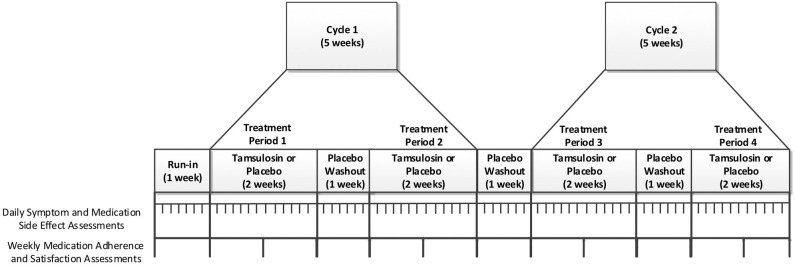
Individual n-of-1 trial.

Participants will start with 1-week open label run-in period where they will use the PERSONAL app to track daily symptoms and side effects while not taking their tamsulosin or any study pills. Based on the pharmacokinetics and expected timeframe of symptomatic relief from tamsulosin (half-life = 14 to 15 h; steady state by the 5th day of daily dosing) (26), all n-of-1 trials will have a total duration of 11 weeks during which participants will complete 2 cycles consisting of a pair of 2-week treatment periods (taking tamsulosin or placebo) separated by 1 week of wash-out on placebo (Figure 2). The order of treatment periods within a cycle will be random (e.g., ABAB, BABA, ABBA, or BAAB) according to pre-filled bubble packs given to participants during their orientation visit. Participants will receive placebo during wash-out periods between treatment periods and cycles, but they will not be aware of the order or duration of treatment periods or cycles to prevent them from self-correlating symptoms to specific treatments.

**Figure 3:**
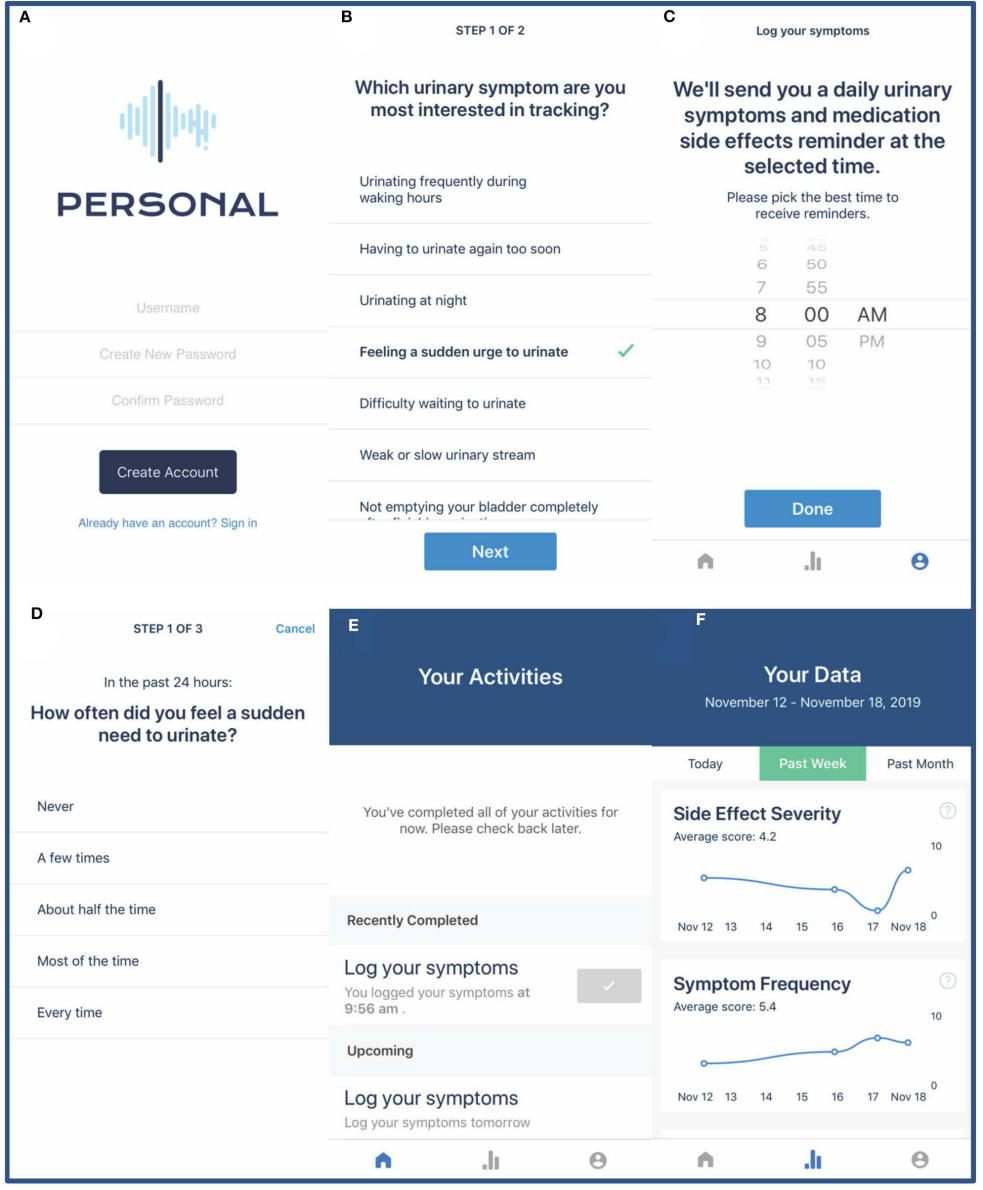
PERSONAL app screenshots. Screenshots demonstrate the PERSONAL app login page **(A)**, selecting urinary symptoms for daily tracking **(B)**, setting a daily questionnaire reminder **(C)**, completing the daily questionnaire **(D)**, the PERSONAL app home page with current and future tasks **(E)**, and graphical representation of participant data **(F)**.

Our team, in collaboration with Overlap Health (https://www.overlaphealth.com/), has developed and tested the PERSONAL app among older men with LUTS due to presumed BPH. The PERSONAL app presents participants with a daily questionnaire to track their individually selected urinary symptoms and medication side effects (Figure 3). All participants will also be presented a global urinary symptom bother question. Depending on how many symptoms and side effects they desire to track, participants will be asked a minimum of 3 and maximum of 5 daily questions for the duration of their n-of-1 trial. At the end of each week, participants will receive additional medication adherence and treatment satisfaction questionnaires administered via the PERSONAL app as well as motivational messages summarizing their progress in the trial. Participants will be able to view a graphical representation of their responses summarized in chronological order for the prior day, week, or month. To maximize adherence to daily questionnaires, participants will be contacted via email or phone if they have completed fewer than 4 daily questionnaires in any week during their n-of-1 trial.

#### Study Completion Visit

Upon completion of their n-of-1 trial, participants will be sent an end-of-study RedCap questionnaire via secure email along with an invitation to schedule a study completion visit with research staff. Research staff will access the participants results using the PERSONAL desktop interface and review results with them during the study completion visit. N-of-1 trial results will be displayed in a series of graphs and text output showing the mean daily urinary symptom, global urinary bother, and medication side effect scores while taking tamsulosin vs. placebo. Within-person treatment effects and confidence limits based on linear mixed effects models (see section Data Analysis below for more detail) will also be shared using patient-friendly graphs and text. We will obtain qualitative feedback on the orientation, PERSONAL app, and usefulness of the data and visualization at the end of study completion visit.

### Assessments and Outcome Measures

#### Feasibility and Acceptability Outcomes

We will evaluate 4 primary outcomes that will be used to determine feasibility and acceptability of a larger future n-of-1 study:

1. Recruitment and retention of 20 eligible men within reasonable timeframe (goal 3–6 months)
2. N-of-1 trial completion rate (goal >70%)
3. Daily questionnaire completion rate (goal >50%)
4. Percentage of participants who describe the PERSONAL app as “extremely helpful” or “very helpful” for deciding whether tamsulosin is an effective medication for them to continue or discontinue (goal >50%).

To calculate the recruitment timeframe, we will log the start date of each individual n-of-1 trial. N-of-1 trials will be considered complete when participants complete the end-of-study questionnaire. Completion of daily questionnaires is tracked by the PERSONAL app along with session duration and distribution of time spent with each component of the app to further quantify user engagement. To characterize the experience of participants using the PERSONAL app, we will ask them at the end of the study to rate the helpfulness of the app from 1 (extremely helpful) to 5 (not at all helpful) across multiple domains based on prior mobile health studies (27). We will also administer an adapted System Usability Scale to characterize usability and functionality of the PERSONAL app (28).

#### Patient-Selected Outcomes

##### Daily urinary symptom severity

Participants will select from daily urinary symptom measures that were adapted from the LURN SI-10 (25). Specifically, we will use 7 of the 10 questions in the LURN SI-10 (including frequency, nocturia, urgency, voiding, and post-micturition symptoms) which have been previously published using 24-h recall periods and were integrated in the PERSONAL app (29). The LURN SI-10 questions excluded from this study refer to urinary incontinence, an exclusion criteria, and bladder pain, a type of LUTS that is not commonly treated with tamsulosin. Specific wording and response options for each question are listed in Supplemental Table 1. For visualization purposes, all responses will be normalized to a scale of 0 (minimal severity or bother) to 10 (maximum severity or bother) to ensure uniform graphical representation.

##### Daily urinary symptom bother

We adapted the American Urologic Association Symptom Index global bother question and changed the recall period from the past month to the past 24 h (30). All participants will be asked “Over the past 24 h, how bothered were you by urinary symptoms?” regardless of which urinary symptoms they are tracking. Responses include “Not at all bothered,” “Somewhat bothered,” “Very bothered,” and “Extremely bothered.”

##### Daily medication side effect bother

To evaluate side effects of tamsulosin, we further adapted the global bother question and will ask participants to quantify how bothered they were by specific perceived side effects. Participants will be asked “Over the past 24 h, how bothered were you by [side effect]?” for each of the medication side effects selected to track during the study orientation visit. Responses rangeD from “Not at all bothered” to “Extremely bothered.” Specific wording and response options for each question are listed in Supplemental Table 1.

#### Investigator-Selected Outcomes

At baseline, we will collect demographic data on age, marital status, race, ethnicity, employment, income, and educational attainment via questionnaire. We will ask participants about their smoking history, alcohol and caffeine intake, and physical activity as well as history of medical conditions, including cardiovascular disease, diabetes, prostate cancer, and prostatitis. At baseline and the end of study, participants attitudes toward deprescribing will be assessed using Revised Patients' Attitudes Toward Deprescribing (rPATD) (31) and health-related quality of life will be assessed using the NIH PROMIS 29+2 Profile (32). At baseline and each week, we will assess self-reported medication adherence and reasons for non-adherence (33) as well as overall satisfaction with current LUTS treatment regimen.

### Data Analysis

#### Sample Size

Based on prior mobile health studies (27, 34–36), we expect to observe a failed primary outcome, such as inability to recruit sufficient participants or reach goal questionnaire and n-of-1 trial completion rates, at least 10% of the time. Therefore, with a sample size of 20 participants, we will have 90% power to observe at least one failed primary outcome during this feasibility study (37, 38).

#### Analytic Plan

Primary outcomes will be assessed as binary variables (e.g., did or did not successfully recruit goal sample size within appropriate timeframe) and the feasibility study will be considered successful if all 4 primary outcome objectives are met. We will describe the primary outcomes as well as baseline demographic and clinical characteristics using percentages, means ± standard deviations for normally distributed variables, and medians with interquartile ranges for skewed variables. The change in secondary and tertiary outcomes from baseline to end-of-study, including LUTS treatment satisfaction, attitudes toward deprescribing, and health-related quality of life, will be evaluated using paired samples *t*-test for continuous measures and McNemar tests for binary variables. We will use multivariable-adjusted linear mixed effects models, with random intercepts and slopes and an unstructured variance-covariance matrix, to estimate variation in daily urinary symptoms or medications side effects. We are aware that there is unlikely to be a sufficient sample size of men who tracked the same urinary symptom or medication side effect needed to calculate valid between-person estimates in this feasibility study, however, participants may have sufficient data to calculate valid within-person variability for each treatment group as well as within-person treatment effects. These estimates will be more accurate and precise in a larger future trial where data from other participants tracking the same symptoms or side effects is incorporated into the linear mixed effects models and contributes to within-person estimates.

### Safety

#### Participant Confidentiality

Data entered into the PERSONAL app will be hosted on the Overlap Health secure environment and will contain no personal health information. To protect participant confidentiality, Overlap Health will only have access to participant study ID numbers. The raw data collected by the PERSONAL app will not be available to other applications. Data transfers will use HIPPA-compliant file encryption (at rest and in transit), secure file transfer (SFTP), Secure Sockets Layer (SSL) for interface data transfers, predefined authentication routes, and a role-based permission system. Questionnaires will be collected electronically via RedCap surveys managed in secure environments behind institutional firewalls. All study staff will be trained in good clinical practice, HIPPA procedures, and participant confidentiality.

#### Data Monitoring

A unblinded Safety Monitoring Committee will be established to review unanticipated or serious adverse events for the duration of the study and will report directly to the University of California, San Francisco Institutional Review Board.

#### Ethics Approval

Ethical approval was granted to our team by the University of California, San Francisco Institutional Review Board for a PERSONAL app pilot study (#19-28557). The feasibility study protocol will build off this prior work and will be submitted for approval by the University of California, San Francisco Institutional Review Board as well as registered on ClinicalTrials.gov once finalized.

## Discussion

The PERSONAL study leverages mobile health technology, n-of-1 trials, patient-selected outcomes, and placebo controls to provide older men with personalized information regarding the benefits and harms of continuing or discontinuing chronic tamsulosin therapy. This study protocol seeks to evaluate the feasibility and acceptability of using the PERSONAL app to conduct a series of n-of-trials. We will collect the data needed to plan a larger future n-of-1 study to provide individualized estimates of patient-selected benefits and harms of chronic tamsulosin therapy among older men with LUTS.

There is likely both undertreatment and overtreatment of LUTS with chronic tamsulosin therapy. Potential contributors to overtreatment include situations where there is minimal or no benefit of long-term treatment (e.g., overestimation of symptomatic relief due to placebo effects or regression to the mean, waning efficacy with longer-term treatment, symptoms refractory to tamsulosin) and situations where harms exceed benefit (e.g., medication side effects, polypharmacy, adverse drug events, drug-drug interactions). Harms from chronic tamsulosin therapy are often insidious; tamsulosin or polypharmacy-related side effects may be attributed to other medications and comorbidities or inappropriately tolerated as a “normal process of aging.” Conversely, men who would benefit from chronic tamsulosin therapy may prematurely discontinue due to misattributed harm (e.g., a mechanical fall in the absence of symptomatic orthostatic hypotension) or perceived lack of benefit due to overlapping conditions (e.g., improved nocturia but persistent insomnia). Since there is currently no recommended minimum or maximum duration of α_1_-blocker therapy for LUTS, n-of-1 trials could be used to personalize LUTS treatments by quantifying both benefits and harms of continuing or discontinuing chronic tamsulosin therapy.

Several observational and small open-label randomized clinical trials provide evidence that discontinuation of chronic tamsulosin therapy will not lead to worsening symptoms in many men. Among 33 men who initially experienced symptomatic improvement with α_1_-blocker monotherapy, mean symptom severity were not increased up to 6 months after unblinded discontinuation and more than two-thirds of men remained off medication (39). Another unblinded study among 75 men with symptomatic improvement after α_1_-blocker monotherapy demonstrated stable symptoms for up to 12 months after discontinuation with only 30% of men requesting re-initiation of treatment (40). Even among men with more severe LUTS who are treated with combined α_1_-blockers plus 5α-reductase inhibitor therapy, both observational and randomized studies have demonstrated no symptomatic progression in the majority of men who discontinue α_1_-blockers but continue 5α-reductase inhibitor monotherapy (41–44). In the largest randomized clinical trial of 230 men receiving combined therapy who were assigned to discontinue either 5α-reductase inhibitor or α_1_-blocker, 74% of men in both groups had no worsening of symptoms after 12 months (45). Despite preliminary evidence that α_1_-blockers can be safely discontinued in men with a wide range of LUTS severity without significant worsening of symptoms, the effects of chronic tamsulosin discontinuation remain unknown and rigorous placebo-controlled studies of α_1_-blocker discontinuation are lacking. We also know that the effects averaged over large numbers of participants in traditional placebo-controlled RCTs do not translate directly to individuals, particularly older adults (18, 21). New approaches to personalized prescribing and deprescribing, such as n-of-1 trials, are needed to determine whether an individual man is receiving more benefit than harm from chronic tamsulosin therapy.

N-of-1 trials have the potential to greatly increase the accuracy and precision with which urologic medications are prescribed for symptomatic conditions such as LUTS. Mobile health technology has lowered many of the barriers to implementing this powerful study design in research, clinical, and non-clinical settings by decreasing the burden of frequent data collection and facilitating data interpretation through instantaneous visualization, however it remains unknown if placebo-controlled n-of-1 trials using patient-selected outcomes are feasible. To address this gap, we will establish the feasibility and acceptability of placebo-controlled n-of-1 trials using the PERSONAL app and patient-selected outcomes among older men with LUTS.

## Data Availability Statement

The raw data supporting the conclusions of this article will be made available by the authors, without undue reservation, to any qualified researcher.

## Author Contributions

SB, BB, AO-O, MS, IS, and SK conceptualized the study and contributed to the study design. SB and CM developed the analytic plan. BB, AO-O, MS, IS, and SK provided administrative, technical, or material support. SB wrote the manuscript and obtained funding. All authors contributed to the refinement of the study protocol and the manuscript. All authors read and approved the final manuscript.

## Conflict of Interest

The authors declare that the research was conducted in the absence of any commercial or financial relationships that could be construed as a potential conflict of interest.
