# Supplementary material for: PERSONAL: Feasibility Study Protocol for Placebo-Controlled, Randomized n-of-1 Trials of Tamsulosin for Lower Urinary Tract Symptoms
Source: Front Digit Health. 2020 Jun 26;2:7. doi: 10.3389/fdgth.2020.00007 (PMC8521798; doi:10.3389/fdgth.2020.00007)
Supplement: Supplementary file 1 [file Table_1.DOCX]

| **Supplemental Table 1.** Daily urinary symptoms and medication side effects questions and responses. | |
| --- | --- |
| **Urinary Symptoms*** | **Response Options** |
| During waking hours today, how many times did you urinate? | 3 or fewer times per day; 4-7 times per day; 8-10 times per day; 11 or more times per day |
| During the day today, how much time typically passed between urinations? | More than 6 hours; 5-6 hours; 3-4 hours; 1-2 hours; Less than 1 hour |
| Last night, how many times did you wake up and urinate? | None; 1 time; 2-3 times; More than 3 times |
| In the past 24 hours: How often did you feel a sudden need to urinate? | Never; A few times; About half the time; Most of the time; Every time |
| In the past 24 hours: Once you noticed the need to urinate, how difficult was it to wait more than a few minutes? | Not difficult; A little difficult; Somewhat difficult; Very difficult; Unable to wait |
| In the past 24 hours: How often was your urine flow slow or weak? | Never; A few times; About half the time; Most of the time; Every time |
| In the past 24 hours: How often did you feel that your bladder was not completely empty after urination? | Never; A few times; About half the time; Most of the time; Every time |
| **Medication Side Effects** | **Response Options** |
| In the past 24 hours, how bothered were you by dizziness or lightheadedness? | Not at all bothered; Somewhat bothered; Very bothered; Extremely bothered |
| In the past 24 hours, how bothered were you by headache? | Not at all bothered; Somewhat bothered; Very bothered; Extremely bothered |
| In the past 24 hours, how bothered were you by erection or ejaculation difficulties? | Not at all bothered; Somewhat bothered; Very bothered; Extremely bothered |
| In the past 24 hours, how bothered were you by decreased libido or sex drive? | Not at all bothered; Somewhat bothered; Very bothered; Extremely bothered |
| In the past 24 hours, how bothered were you by runny nose? | Not at all bothered; Somewhat bothered; Very bothered; Extremely bothered |
| In the past 24 hours, how bothered were you by fatigue or excessive tiredness? | Not at all bothered; Somewhat bothered; Very bothered; Extremely bothered |
| In the past 24 hours, how bothered were you by insomnia or difficulty sleeping? | Not at all bothered; Somewhat bothered; Very bothered; Extremely bothered |
| In the past 24 hours, how bothered were you by diarrhea? | Not at all bothered; Somewhat bothered; Very bothered; Extremely bothered |
| In the past 24 hours, how bothered were you by constipation? | Not at all bothered; Somewhat bothered; Very bothered; Extremely bothered |
| In the past 24 hours, how bothered were you by nausea? | Not at all bothered; Somewhat bothered; Very bothered; Extremely bothered |
| In the past 24 hours, how bothered were you by weakness? | Not at all bothered; Somewhat bothered; Very bothered; Extremely bothered |
| In the past 24 hours, how bothered were you by back pain? | Not at all bothered; Somewhat bothered; Very bothered; Extremely bothered |

* Adapted from daily urinary symptom assessments used by Flynn et al.(29)
